# Addressing Challenges of Macrocyclic Conformational Sampling in Polar and Apolar Solvents: Lessons for Chameleonicity

**DOI:** 10.1021/acs.jcim.3c01123

**Published:** 2023-11-09

**Authors:** Xuechen Tang, Janik Kokot, Franz Waibl, Monica L. Fernández-Quintero, Anna S. Kamenik, Klaus R. Liedl

**Affiliations:** † Department of General, Inorganic and Theoretical Chemistry, University of Innsbruck, A-6020 Innsbruck, Austria; ‡ Department of Chemistry and Applied Biosciences, ETH Zürich, 8093 Zürich, Switzerland

## Abstract

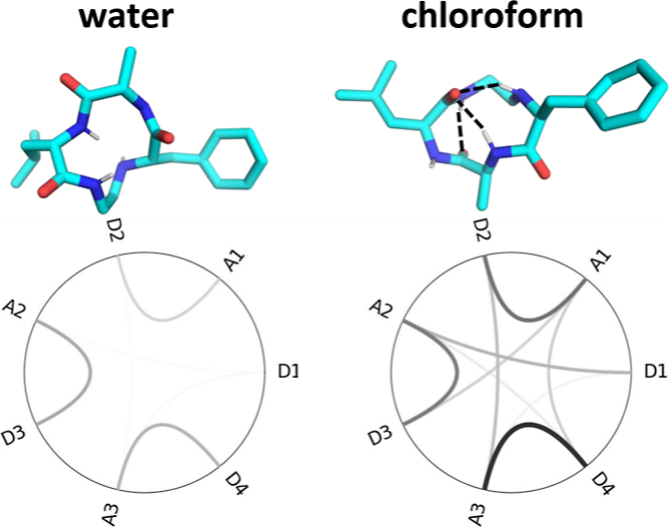

We evaluated a workflow to reliably sample the conformational space of a set of 47 peptidic macrocycles. Starting from SMILES strings, we use accelerated molecular dynamics simulations to overcome high energy barriers, in particular, the cis–trans isomerization of peptide bonds. We find that our approach performs very well in polar solvents like water and dimethyl sulfoxide. Interestingly, the protonation state of a secondary amine in the ring only slightly influences the conformational ensembles of our test systems. For several of the macrocycles, determining the conformational distribution in chloroform turns out to be considerably more challenging. Especially, the choice of partial charges crucially influences the ensembles in chloroform. We address these challenges by modifying initial structures and the choice of partial charges. Our results suggest that special care has to be taken to understand the configurational distribution in apolar solvents, which is a key step toward a reliable prediction of membrane permeation of macrocycles and their chameleonic properties.

## Introduction

Macrocycles are promising candidates for targeting difficult binding sites, such as protein–protein interfaces and shallow, solvent exposed surfaces on intra- and extracellular targets.^1−11^ Their unique binding abilities are thought to originate from ring constraints and peptide bonds in the case of peptidic macrocycles, which are hard to mimic with small molecules.^12−20^ However, their large sizes and high polar atom counts hinder macrocycles from crossing membranes, such as small molecules. Nevertheless, several macrocycles exhibit decent membrane permeability due to the so-called chameleonic behavior.^21−36^ This comprises exposing polar atoms in an open state in polar solvents and minimizing polar surfaces in a closed state in apolar environments, such as a membrane. The latter can be achieved by shielding of the polar surface with bulky hydrophobic fragments or formation of intramolecular hydrogen bonds (IMHBs); however, the chemical nature of chameleonicity is under debate and its quantitative measurements for cell permeability predictions remain partially unclear.^24,37−43^ More studies on conformational preferences in different environments are needed to fully understand the mechanism of chameleonic membrane passing and the solvation of macrocycles in polar and apolar environments.^22,44−47^ Due to their unconventional conformational changes, such as peptidic bond inversions,^31,48−53^ dynamic patterns of the dense IMHBs,^24,40^ and restrained ring deformations,^54−61^ short classical molecular dynamics (MD) simulations hardly capture different conformational states. Exhaustive sampling of the conformational spaces remains challenging.^54,62−64^

MD simulations can improve the understanding of the conformational distribution of peptidic macrocycles, as is recently summarized by Damjanovic et al.^54^ Especially, various enhanced sampling techniques have been explored to cover their intrinsic flexibility.^54^ In combination with experimental techniques like the EPSA, a measure of polarity^65^ and NMR,^66−74^ these simulations capture structural descriptors and key dynamic steps associated with pharmacological properties of interest in individual macrocyclic subsets, for example, intramolecular hydrogen bond rearrangements and anchoring to the apolar membrane for cell permeability as well as exposure of key polar atoms for binding events. Some of the challenges for macrocyclic simulations are sampling efficiency,^62−64^ force field settings,^61,75^ and incorporation of solvent effects.^54,54,76^ For example, partial charges are often assigned with single starting structures despite their importance in apolar environments due to the small dampening by low dielectric constants.^77^

Enhanced sampling methods aim to overcome the kinetic and potential energy barriers that are hard to overcome with classical MD simulations (cMD).^78−96^ These methods are generally split into two groups, global biasing methods boosting the kinetic or potential energy^87,97,98^ and pathway-dependent/local biasing methods boosting predefined features of the system called collective variables (CVs).^80,90,99^ For the latter, the space sampled depends on the CVs, which often requires expected directions of system evolution as input. Thus, they need accurate theoretical assumptions or experimental guidance to minimize the distortion of the conformational space and ensure time-efficient sampling.^96,99−103^ On the other hand, global biasing methods boost all possible evolutions of the system simultaneously, offering more complete views on the conformational spaces.^78−80,89−92,96^ Accelerated MD (aMD) is one of the global potential energy flattening/smoothening methods.^82^ aMD speeds up high-energy conformational transitions by softening constraints imposed by dihedral torsions and other potential energy contributors. Therefore, it samples distant conformations separated by high-energy barriers, hardly accessible with cMD of typical length. In fact, a benchmark on peptidic systems with 2–159 residues showed that aMD can indeed overcome torsional barriers and speed up the sampling by 3 orders of magnitude.^104^ aMD trajectories can also be reasonably reweighted to recover the original free energy landscape with sufficient sampling.^105−108^

**Figure 1:**
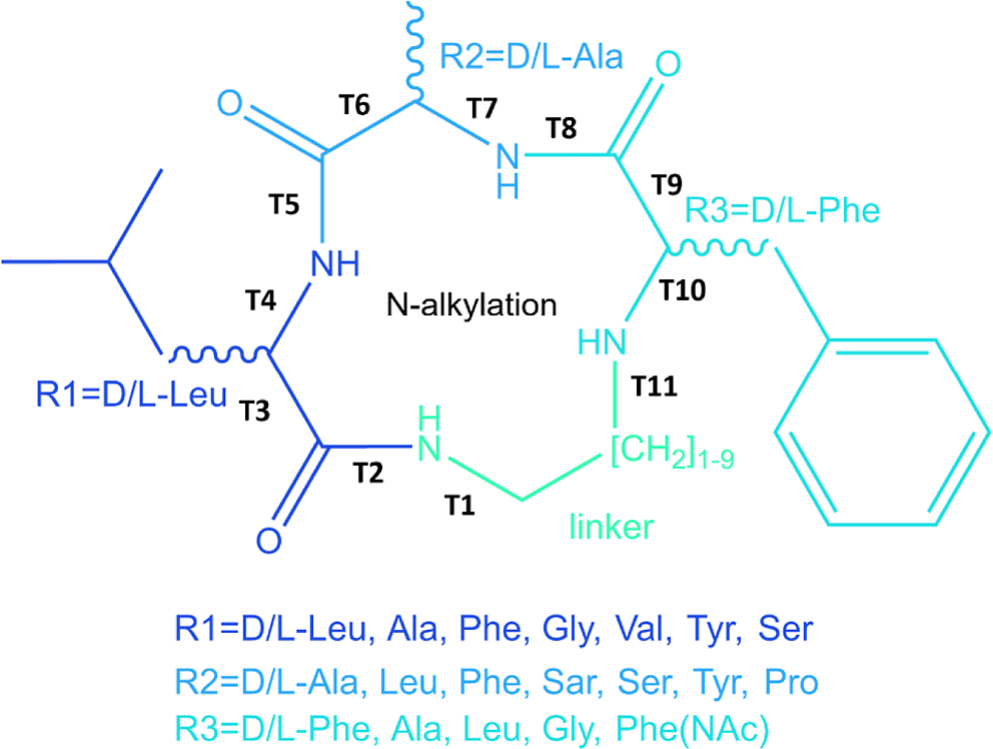
Modeled macrocyclic systems taken from Le Roux et al.^109^ All structures comprise three (d/l-) amino acids, which form a ring with an alkyl linker. Linker size as well as types, positions/orders, and stereochemistry of the amino acids had been tested for their influence on the cell permeability. Some of these semipeptidic macrocycles can contain additional N-alkylation and acetylation. T1–11 are torsions included in the principle component analysis (PCA).

Here, we present an aMD^82^ sampling study on a large systematic set of 47 peptidic macrocycles with backbone modifications from Le Roux et al.^109^ The studied compounds comprise a large variety of structural modifications, for example, linker length, bulky side chains, rigidification by proline, additional side chain polar atoms, stereochemistry, and N-methylation, from a common scaffold (Figure 1). We employ a pre-established protocol by Kamenik et al.^110^ This systematic test set enables a thorough comparison throughout the entire series, while the large variety of modifications covered intrinsically test the validity of a sampling protocol. In the previous works,^15,110^ we sampled in explicit water and membrane mimicking chloroform, testing calculated transfer free energy from reweighted ensembles with experimentally measured cell permeability.^111^ In this study, we tested the efficiency and transferability of this protocol considering different protonation states and in different solvents. Intramolecular hydrogen bond analyses are applied to track the root of conformational changes and solvent effects. We report potential challenges and suggestions for future macrocycle sampling studies.

## Methods

To prepare the initial structures, 3D conformations were generated from the Simplified Molecular Input Line Entry System (SMILES)^112^ provided by Le Roux et al.^109^ with RDKit^58,113^ and protonated in the molecular operating environment (MOE)^114^ with the “wash” function at pH 7.4. For macrocycles 1 and 2, we had to apply the protonate 3D in MOE at pH 5.4 to prepare the charged forms. The neutral forms are generated without additional protonation of the ring amine-N. To generate the topology file for further geometric optimization, we assigned partial charges to the macrocycles by the restrained electrostatic potential (RESP)^115^ approach using the HF/6-31G* basis set with an optimized structure in Gaussian 09.^116^ Meanwhile, averaged charges were calculated from 10 randomly generated structures by the experimental-torsion-knowledge distance geometry (ETKDG version 3)^58^ module of the RDKit. We assigned atom types with antechamber^117^ and parametrized other potential energy terms, e.g., van der Waals and torsions with the ff14SB^118^ and GAFF^119^ by tLEaP in the AmberTools 19 package.^120^ Finally, we obtained the topology files and coordinates of the systems solvated in TIP3P water,^121^ chloroform,^122^ and dimethyl sulfoxide (DMSO)^122^ at 12 Å wall distance with the tLEaP.

For the structural ensembles, we used the aMD protocol adapted from Kamenik et al.,^110^ which comprises standard preparation in explicit water and chloroform,^123^ followed by 1 μs of aMD with dual boost to generate structural ensembles in AMBER20^124^ with the PMEMD.^125,126^ We apply the SHAKE algorithm^127^ to restrain the hydrogen movements, which allows a larger time step of 2 fs for water and DMSO simulations. We chose the boosting parameters according to Pierce et al.^108^ for water and chloroform. We set dihedral boosts according to number of freely movable backbone dihedrals and potential energy boosts to all atoms in the systems, as is described by Kamenik et al.^110^ Because DMSO has more internal degrees of freedom than water and chloroform, we increased the potential energy threshold for boosting to be 0.56 kcal/mol times the number of atoms above the unbiased potential energy in that solvent. To recover the unbiased free energy surfaces and distribution of the IMHB, we applied Maclaurin reweighting^128,129^ to the 20th order.

To analyze the trajectories, we calculated the 2D RMSD with CPPTRAJ^130^ and checked macrocycle conformations with PyMOL.^131^ We plotted the conformational space with the first two principal axes from the principal component analysis (PCA) of sine and cosine of the 11 dihedrals common to all macrocycles in the series to visualize the general distribution of the clusters and the population shifts. To investigate if the similar PCA projection indeed represents similar conformations in polar solvents, we clustered the conformations with the PCs plotted. We first clustered the conformations of the macrocycle 1 with its charged form in water by K-means, a distance-based clustering method implemented in CPPTRAJ^130^ with six centroids, to select representative conformations near the deepest free energy minima. We visualized the cluster representative of the first two clusters (named cluster 1 and cluster 2 for the following) and compared them with representatives from other macrocycle 1 samplings. To assess the similarity of conformations in polar solvents, we selected the closest cluster representatives to cluster 1 and cluster 2 in polar solvent for comparison. In the absence of an adequate cluster, as is the case for DMSO cluster 1, we chose the closest frame to the aqueous cluster representative. For sampling in chloroform, where the clusters are completely different, we simply picked the first two cluster representatives for visualization.

We test the reproducibility of the workflow by checking whether samplings starting from two different initial structures converges. We run the test on 10 out of 47 macrocycles, whose 2D RMSD show slower transitions and thus are the most challenging in terms of sampling. We start a second aMD sampling with the ETKDG structure projected farthest away from the initial structure on the PCA, out of three ETKDG structures (Supporting Information, Figure 4). We reassigned the partial charges starting from the new structure and went through the entire workflow from standard preparation. We define the reproducibility test as passed if the PCA of the initial sampling resembles that of the repetitive sampling from the second starting structure. We then picked out the only molecule, that is the macrocycle 35 (Supporting Information, Figure 7), whose conformational space failed to converge for samplings with different starting structures to conduct further convergence issue investigations. For the IMHBs between the carbonyl group and NH (cutoff distance at 3.5 Å, suggested by the experimental NMR observations of the short-range IMHB^109^), we adapted the GetContacts_analysis^132^ associated tools to form an in-house script for contact maps. To analyze intramolecular hydrogen bond contribution for the PAMPA cell permeability measurements, we set the angle cut off to 90° to capture the unconventional short-range intramolecular hydrogen bond emphasized by the NMR method^133^ applied in original study^109^ and limit the contribution of low energy contacts. We use results from the aqueous phase and chloroform for aMD to better mimic the PAMPA condition. To evaluate the convergence of the trajectory beyond the 2D RMSD, we also split the trajectory in two and compared the contact map as well as the PCAs of the split halves.

## Results

### Conformational Spaces Explored by aMD Converge

**Figure 2:**
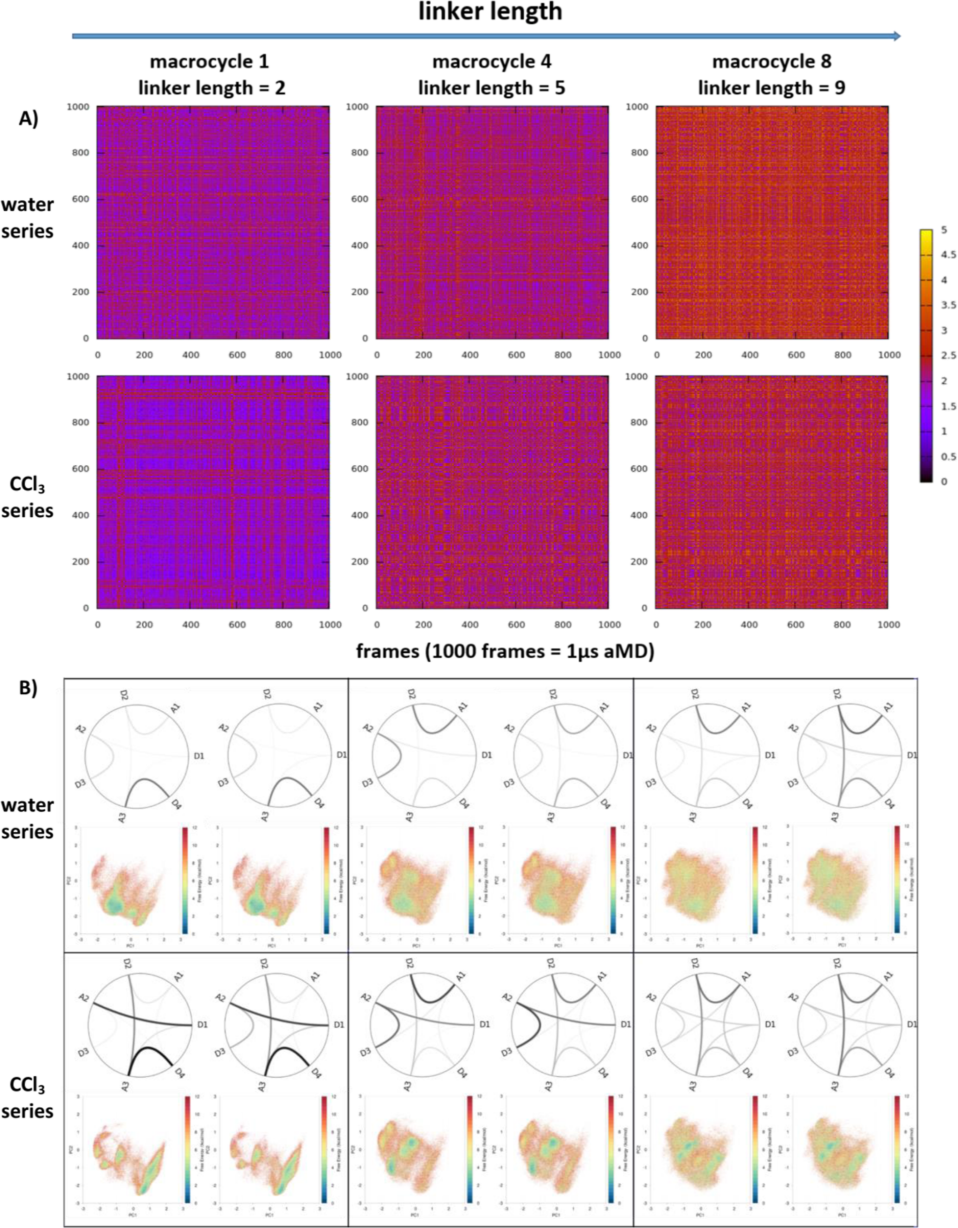
Convergence check of 1 μs aMD simulations in water and in chloroform. (A) The two-dimensional root-mean-square deviation (2D-RMSD) plots are based on all heavy atoms of entire simulations. Dark purple shows lower RMSD, i.e., similar conformations in two frames compared, while light yellow shows higher RMSD, i.e., different conformations in compared frames. Conformational changes are more frequently sampled in water than in chloroform (with the same boosting parameters). Sampling is more challenging in chloroform due to less dampening of electrostatic interactions with a lower dielectric constant. From left to right linker length gradually extends from the minimum two carbon atoms to the intermediate five carbon atoms and the maximal nine carbon atoms. Sampling is more efficient for more flexible macrocycles with longer linkers; however, it converges within the simulation time even for the most rigid systems with the shortest linker of two carbons (in CHCl_3_). (B) Contact maps of intramolecular hydrogen bonds and PCAs based on common torsions for the first 500 ns (left of each block) and the second halves (right of each block). Both contacts shaping conformational distributions and the free energy surfaces show good convergence.

To evaluate the convergence of our simulation, we plot the 2D RMSD (Figure 2A) to look into the similarity between each single frame with the rest of the trajectory. To further confirm the convergence in the conformational distribution, we investigate both the driving force of the distribution depicted in contact maps and the end points depicted by the PCA of common torsions of split trajectories (Figure 2B).

We show the frequency of conformational changes and convergence of the samplings by 2D RMSD plots (Figure 2A). Frames with simulation time intervals of 1 ns are compared to each other within the same trajectories. Depending on the RMSD of macrocycle conformations from the two frames, the cross sections are colored purple for low RMSD, that is, similar conformations, and orange for high RMSD, that is, dissimilar conformations. Thus, the more frequent the color changes are, the faster the conformational changes occur (and the more efficient the sampling is). For example, macrocycles 8 with the longest linker among the three has the most frequent conformational changes in water, while macrocycle 1 with most restraining linker of minimal length has the least conformational changes in chloroform (Supporting Information, Figure 5). Sampling in water is more efficient than that in CHCl_3_ (Figure 2A) as more different structures are sampled within a shorter simulation time. Also, longer linkers result in more flexible molecules as the conformations change more frequently with the increase of linker length. However, even for the most rigid system, that is, macrocycle 1 in CHCl_3_ with the shortest linker, distant conformations are excessively sampled; its conformational space is thoroughly explored. Moreover, its frequency of visiting different conformations stabilizes within the sampling time, that is, the trajectory converges. This is also supported by similar contact maps of the intramolecular hydrogen bonds and PCAs of common torsions between the first and second halves of the trajectories (Figure 2B). Convergence of sampling at different time points can also be seen with PCA of the most rigid system, that is, macrocycle 1 in chloroform colored by frame number (Supporting Information, Figure 6).

Contact maps derived from the first halves of the simulations resemble those of the second halves (Figure 2B). Major IMHBs, shown with dark thick lines, remain unchanged between two halved trajectories. However, contacts only present in limited number of frames shown in gray thinner lines are more sensitive to the relative free energy of single frames for reweighting. Consequently, these less frequent IMHBs may fluctuate slightly between maps from different trajectories with reweighting. Actually, the trajectories before reweighting converge even better and could encourage further splits. Overall, the IMHB patterns and especially major contacts remain constant and converge with the split trajectories.

Visualization of the conformational space also supports convergence among split trajectories (Figure 2B). Distribution of structural clusters as shown by PCAs of common dihedrals superpose between two splits of the same trajectory. Similar to the contact maps, individual spilt plots are slightly noisier than projection of whole trajectories with reweighting. This can result from even less sampling of the high free energy regions in individual splits. Larger free energy fluctuations among smaller number of frames which hold fewer stable conformations can be a major contributor to higher noise level in split trajectories. However, the density distribution and especially the energy minima remain superposing between the first and second half of each trajectory. This result confirms the convergence of density distribution or probability of presence for structural ensembles.

### Sampling is Not Limited by the Starting Structures

We visualize the conformational distribution with the PCA of the 11 common torsion angles of all systems simulated in water and chloroform. The space sampled is independent of the starting conformation. For macrocycle 1, the starting conformation is located completely outside the energetically favored area, for macrocycle 4, in between the low free energy regions, and for macrocycle 8, near the most populated regions. Despite beginning from those points, the sampling is not limited by their locations. Macrocycles also extend the conformational space covered as the linker length grows in all different solvents and protonation states, suggesting that longer linkers result in more flexible macrocycles.

Solvent conformations are similar for polar solvents, even in different protonation states. The major difference occurs in apolar solvent, where conformational distributions become more restrained. Conformational spaces in water resemble those in DMSO. Surprisingly, the charge state in water only slightly shifts the density distribution, leaving the overall space covered nearly identical to those of the neutral ones. Free energy landscapes change drastically in chloroform, and favorable spaces are more restrained and less well connected compared to the polar ones.

### Similarity in Intramolecular Hydrogen Bond for Systems with Similar Conformational Spaces

**Figure 3:**
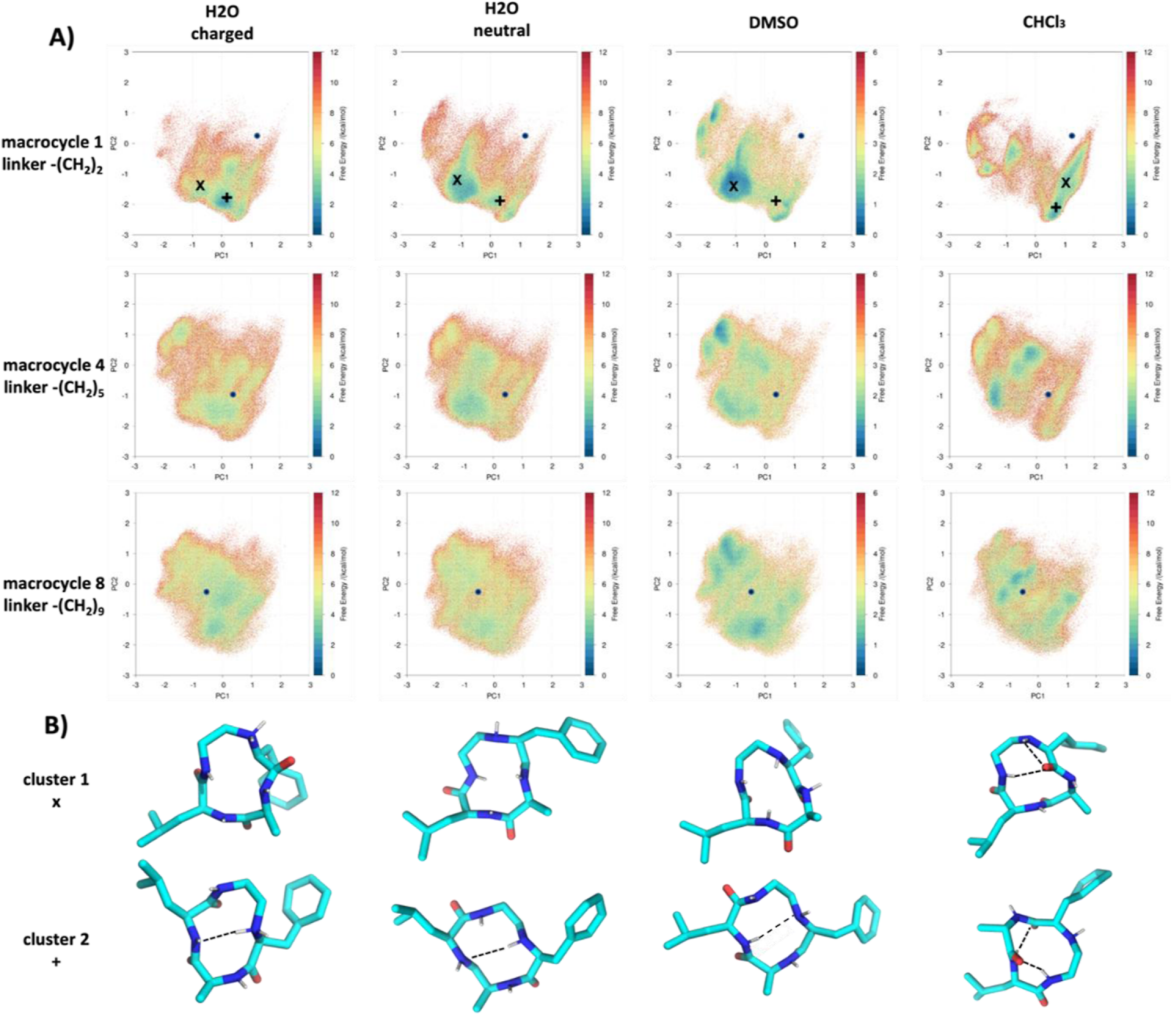
Reweighted conformational distribution of exemplary macrocycles (with extending linker lengths) in various protonation states and solvents. (A) Conformational spaces depicted by the first two principal components of the combined torsional space PCA. The dark blue area represents energetically favored conformational space, while red dots represent high energy conformations. The white space is not sampled. The initial structures (generated by the RDKit) are represented by dark blue dots (they can be located outside the sampled area, e.g., for macrocycle 1, in high free energy zones in between popular clusters, e.g., for macrocycle 4, or near the energy minima, e.g., for macrocycle 8). However, the quality of the starting structure does not seem to affect the conformational space sampled. For all macrocycles, the protonation state has surprisingly limited impact on the coverage of conformational ensembles in water. Meanwhile, conformational spaces in water strongly resemble those in DMSO. (B) Conformations of macrocycle 1 at cluster centers positions depicted in (A).

**Figure 4:**
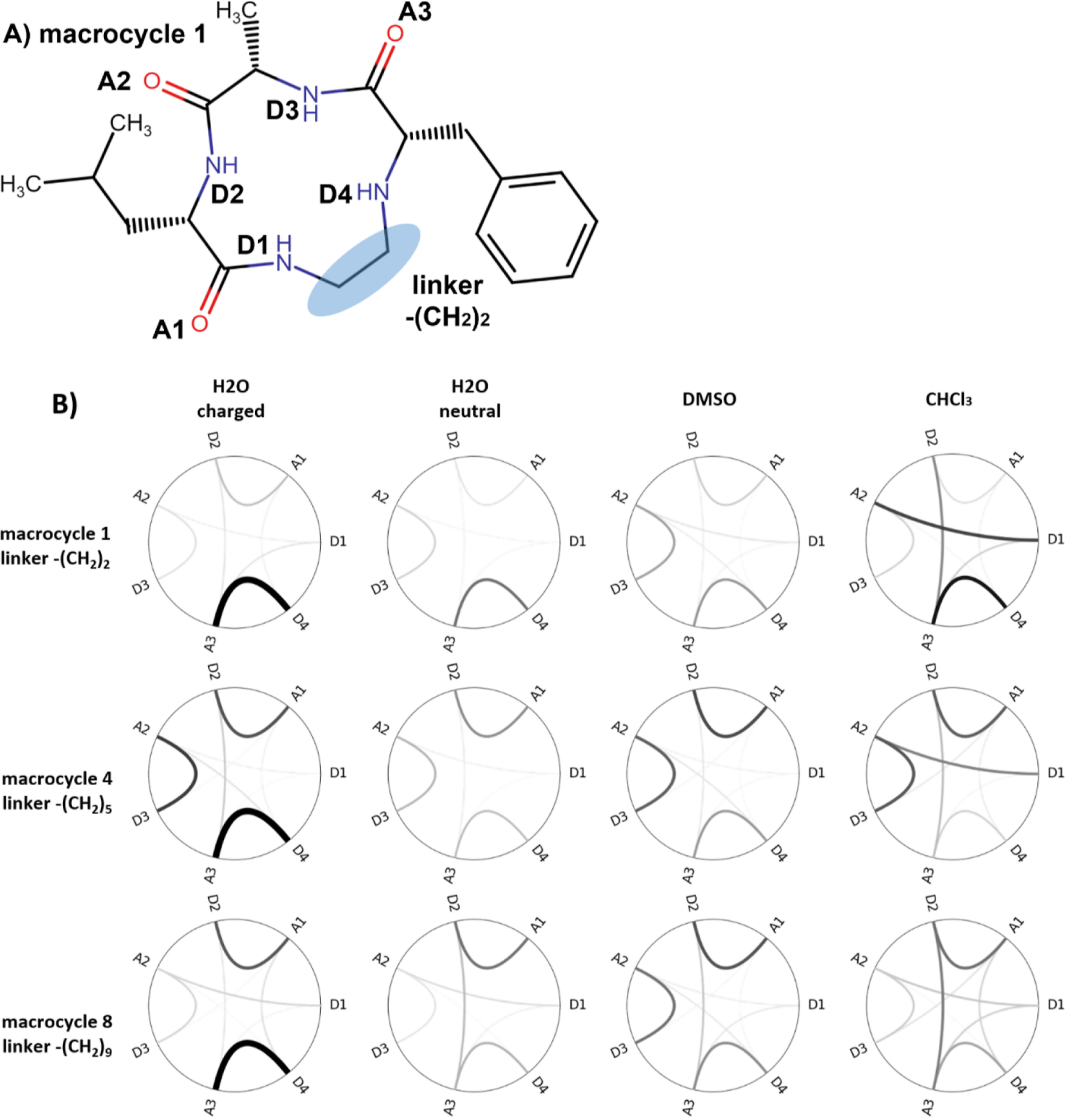
(A) Linker, IMHB donors, and acceptors mapped to macrocycle (B) reweighted intramolecular hydrogen bond (IMHB) patterns as a function of linker length, protonation state and solvent for systems shown in Figure 3. The darkness of lines connecting hydrogen bond donors with acceptors shown in part A reflects the probability of hydrogen bond formation. Intramolecular hydrogen bond patterns are consistent with the conformational spaces in Figure 3 Intramolecular hydrogen bond formations are similar in polar solvents, mostly composed of short-distance IMHBs which appear more frequently as the linker length and flexibility grow. However, intramolecular hydrogen bond patterns in chloroform drastically differ from those in polar solvents by more prominent long-distance IMHBs.

To search for the driving forces of the conformational changes and better characterize chemical features of the conformational spaces sampled, we plot the intramolecular hydrogen bond patterns in Figure 4. Individual IMHBs are represented by lines connecting interaction partners, with the darkness of lines reflecting frequencies of occurrences. The similarity of intramolecular hydrogen bond patterns correlates well with that of the conformational distributions. Intramolecular hydrogen bond patterns of all systems in polar solvents are dominated by short-range IMHBs among neighbors (Supporting Information, Figure 1), more flexible molecules with longer linkers mostly increase probabilities of existing intramolecular hydrogen bond formations systematically. Intramolecular hydrogen bond patterns of the neutral forms in DMSO strongly resemble those in water, while the charged forms introduce slight shifts among short-range IMHBs, for example, increase the probability of an existing intramolecular hydrogen bond involving the charged D4, similar to the mild population shifts in previous PCA plots (Figure 3). However, the CHCl_3_ systems form distinctive patterns for each member without obvious trends. Also, they form much more long-range IMHBs compared to their polar solvent system analogues.

### Intramolecular Hydrogen Bond Distribution from aMD Matches the PAMPA Results

Intramolecular hydrogen bond donor contribution from aMD correlates well with the PAMPA results.^109^ Interestingly, for macrocycles with the same side chains, inhibitions of different intramolecular hydrogen bond donors can result in either increased or decreased PAMPA–log *P*_e_ values, that is, promotions or reductions of the cell permeability. The most significant changes in PAMPA–log *P*_e_ are caused by D4 inhibitions, with macrocycles 26 and 29 reducing cell permeability by three- to fourfold. Another important change is the methylation of D1 in macrocycle 28, which decreases the PAMPA–log *P*_e_, that is, increases the cell permeability by a factor of 2. In terms of the aMD investigation of the donor effects, D4 is the most important intramolecular hydrogen bond donor in chloroform and encounters the biggest differences between apolar and polar phases. In contrast, it is D1 that plays a similar role in the original NMR measurements. Meanwhile in aMD, D1 makes the dominant intramolecular hydrogen bond contribution in the polar phase but appears to be absent in NMR’s DMSO measurements.

### Partial Charges Derived from High Free Energy Initial Structures Can Limit the Sampling of Rigid Macrocycles

We observe one macrocycle out of the entire collection of 47 macrocycles to have a convergence issue in chloroform (Figure 6). Conformational spaces sampled with partial charges derived from the initial structure 1 differ from different starting structures in CHCl_3_. Areas covered are similar in water for both initial structures in different protonation states. In CHCl_3_, sampling with partial charges derived from initial structure 2 results in almost identical free energy landscapes, despite different starting structures. However, the landscapes with partial charges derived from initial structure 1 are completely different for two trajectories starting from different initial structures. Actually, conformational spaces sampled in two separated trajectories only partially overlap with each other, indicating incomplete sampling by this set of partial charges. Initial structure 1 occupies a high free energy state, with the polar NH group between the phenylalanine and the linker approaching a nonpolar Cα inside the ring. The partial charges derived from this physically rather disfavored state seem to limit the sampling efficiency.

**Figure 5:**
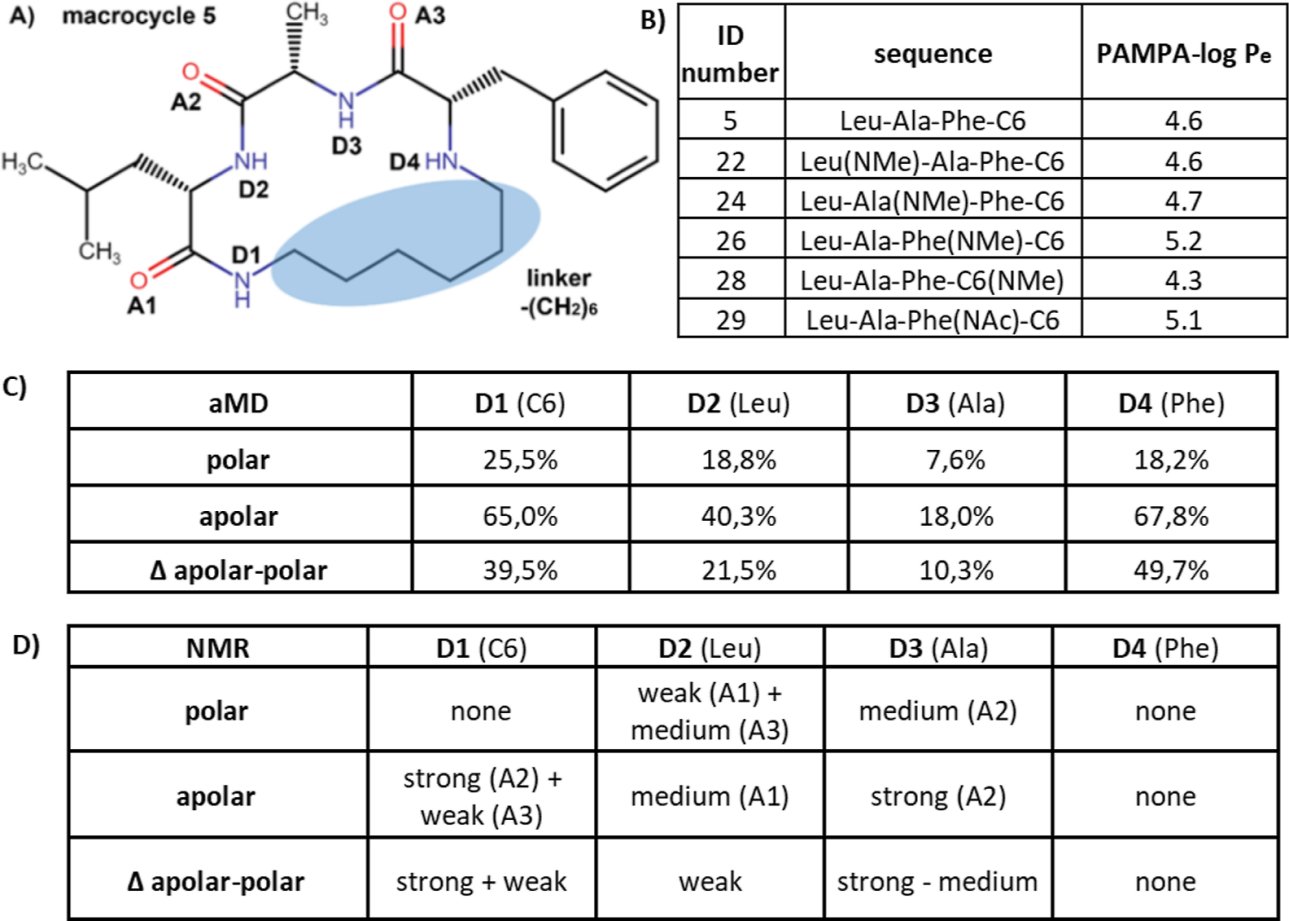
Intramolecular hydrogen bond distribution from aMD reflects experimental PAMPA variations.^109^ (A) IMHB components mapped onto the stem structure, i.e., macrocycle 5. (B) Experimental PAMPA values for N-alkylated variants from Le Roux et al.^109^ (C) Contribution of intramolecular hydrogen bond donors in percentage in polar and apolar solvents from aMD of macrocycle 5 with average charge. (D) Contribution of intramolecular hydrogen bond donors in polar and apolar solvents from the original NMR measurements.^109^

**Figure 6:**
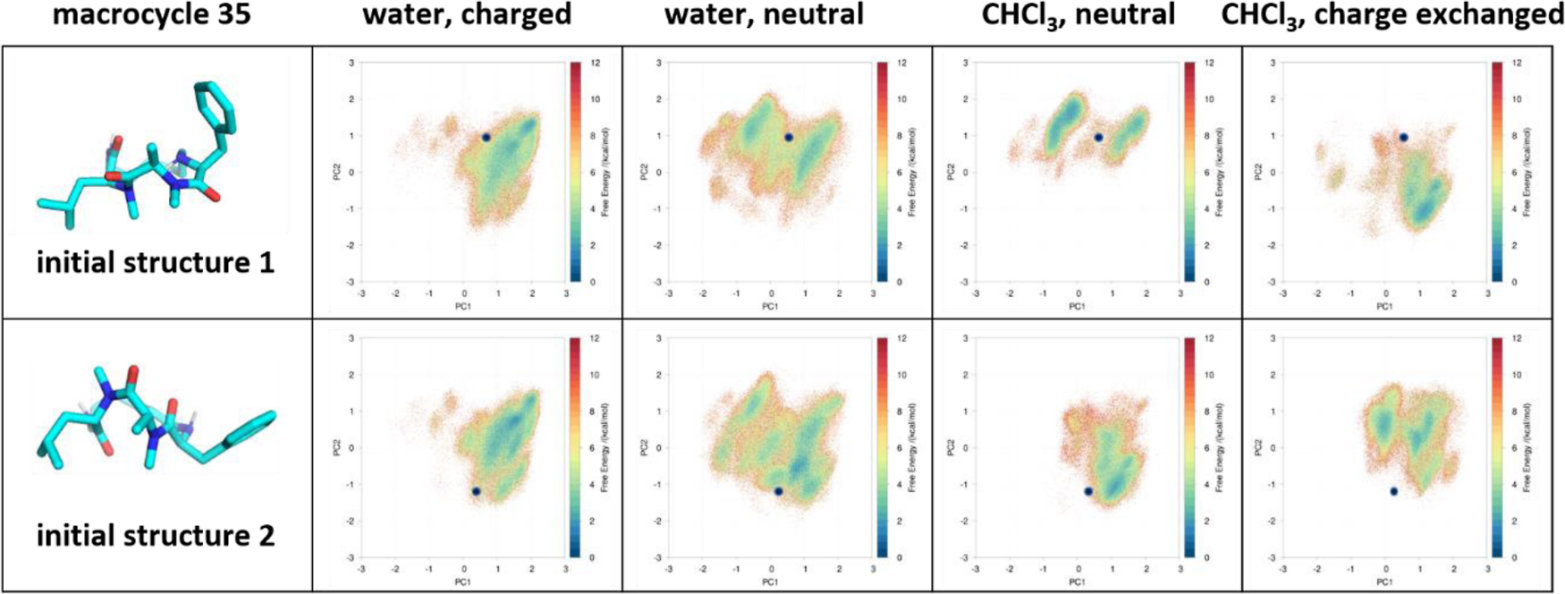
Reweighted conformational spaces of macrocycle 35 with partial charges assigned by single conformation, which is the most challenging to sample. Initial structure 1 has a polar hydrogen on D4 approaching a nonpolar Cα inside the macrocyclic ring, while the initial structure 2 looks more favorable with all polar atoms pointing outside the ring. Conformational distributions of the macrocycles in water look similar to two sets of partial charges derived from different initial structures. However, sampling in chloroform is limited by the starting structures, i.e., by the partial charges derived from the initial structures.

### Using Averaged Partial Charges Restores the Sampling Efficiency, Independent of Starting Structures

**Figure 7:**
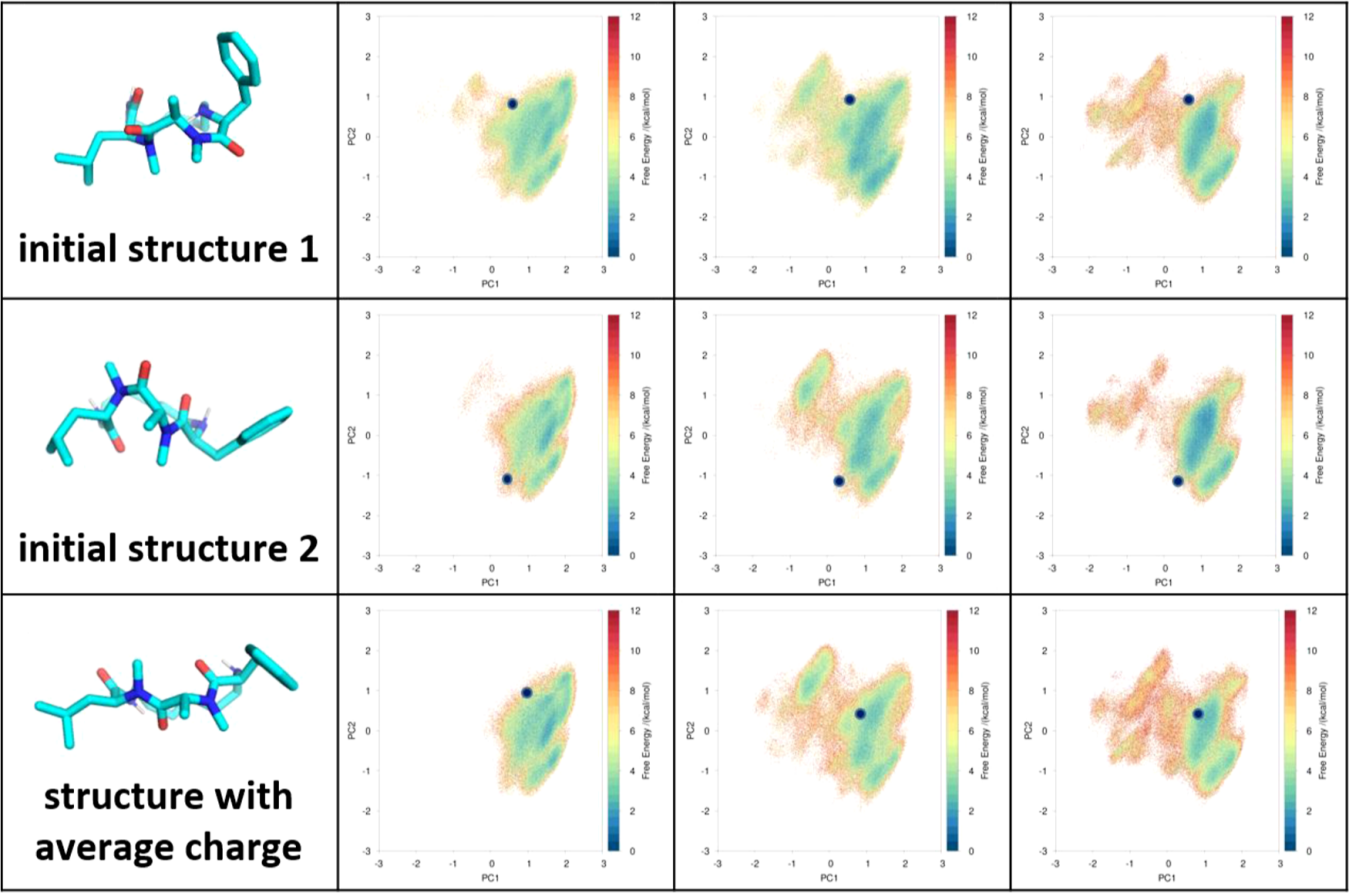
Reweighted conformational spaces of macrocycle 35 with averaged partial charges in different solvents from initial structures in Figure 5 and another randomly generated structure. The partial charges obtained by averaging RESP calculations of 10 ETKDG structures limit the bias of individual structures and remove the constrain of the initial structures on sampling. Conformational distributions in both water and chloroform converge independent of the starting structures with the averaged partial charge.

We show that averaged partial charges solve the convergence issue (Figure 7). By averaging the charges among 10 ETKDG structures, the average charge enables comparable sampling for trajectories starting from different structures. Despite the large differences in starting conformations and their projections into the PCA space, conformational distributions are nearly identical for systems with different protonation states in water and even in chloroform.

### Longer Linker Extends the Conformational Space

**Figure 8:**
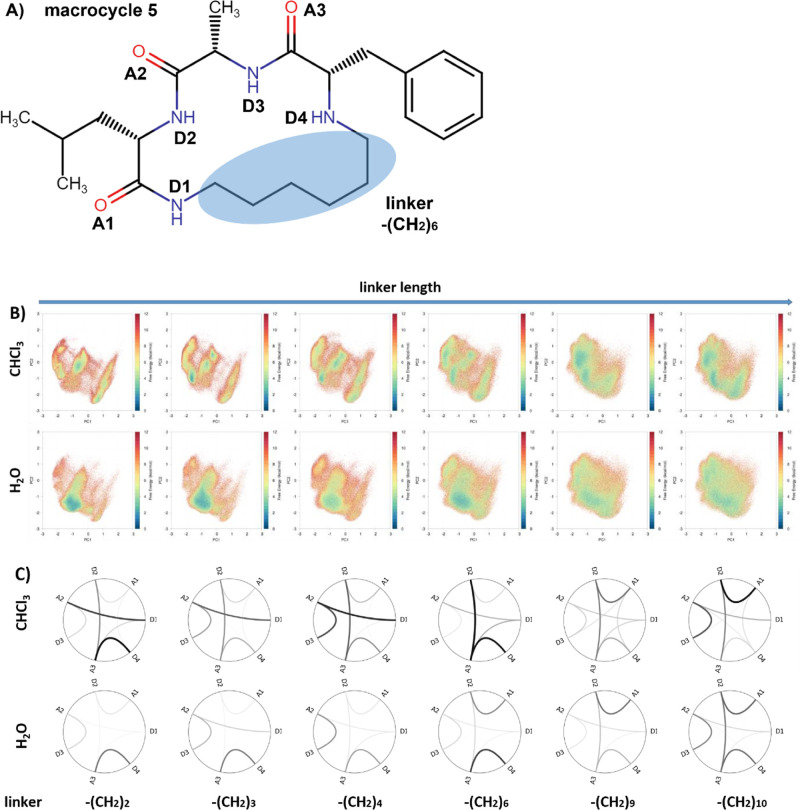
Reweighted conformational space and intramolecular hydrogen bond pattern as functions of linker length and polarity of the solvents. (A) Intramolecular hydrogen bond components and the linker mapped onto the stem structure, i.e., macrocycle 5. (B) From left to right, macrocycles with longer linker extend the spaces sampled both in water and in chloroform. However, the conformational space is more variable in chloroform than in water. (C) Intramolecular hydrogen bond patterns in water are similar for macrocycles with rings of different sizes, although more flexible macrocycles with longer linkers tend to increase the frequency of the same short-range IMHBs and form slightly more long-range IMHBs. On the other hand, intramolecular hydrogen bond patterns in chloroform vary drastically as the linker extends and the long-range IMHBs are more frequently formed than in water.

We investigate the linker effects as an exemplary application of the workflow. In Figure 8, the evolution of the free energy surface and intramolecular hydrogen bond patterns against the linker length for selected macrocycles in the linker series with averaged partial charges are shown. As the conformational spaces are similar in polar solvents, we investigate conformations of the unprotonated macrocycles in water and chloroform to study effects of polar and apolar solvents. As the polar solvents result in similar free energy surfaces, only aqueous systems and membrane mimicking chloroform systems are shown. The systems in both solvents explore larger spaces as the linker grows; however, conformational distributions in polar and apolar solvent are rather distinctive. In water, the PCAs evolve rather systematically, with probability densities extending gradually from the lower left corner to almost the entire possible conformational space. However, conformational distributions are more variable and distinctive for systems in chloroform. The spaces sampled are more limited and show strong free energy preferences over the overall available space. Moreover, the density center shifts irregularly as the linker length extends.

IMHBs show similar trends in variations (Figure 8c). The intramolecular hydrogen bond patterns in water stay similar for all systems in the series, they mainly cover all possible short-range IMHBs. As the linker extends, the probability of short-range intramolecular hydrogen bond formation increases systematically and the long-range IMHBs are occasionally visited although they are nearly negligible compared to the short-range ones, especially for macrocycles with short to intermediate linker lengths. In CHCl_3_, the picture is drastically different and becomes more interesting. Long range IMHBs are formed very early on with short linkers and last throughout the series. However, each individual chloroform system has its preferred long- or short-range IMHBs without extensively sampling all possible pairs. Changes with the intramolecular hydrogen bond patterns in CHCl_3_ are less systematical or predictable.

## Discussion

Convergence is an important quality of MD simulation. It ensures the stability of observables extracted from the trajectory.^134,135^ A well converged trajectory with good sampling of the conformational space provides constant probability distribution of conformational clusters^136^ and reliable estimation of properties for macrocycles, for example, intramolecular hydrogen bond distribution, atomic surface exposure, and their changes between polar and apolar phases. These observables can be compared with experimental measurements to assess the quality of the simulation or applied to aid the design of macrocycles with desired properties,^137,138^ such as improved permeability or exposure of atoms key to binding.^5^ A simulation exploring space beyond the vicinity of a single starting point and converging with different starting conformations offers a dynamic view of conformational distribution in solution. It reveals distributions and interchanges of clusters with the desired properties. With more abstract analyses on driving forces of these conformational distributions and property changes such studies can contribute to the design and fine-tuning of target molecules.^40,137^

Previous simulation works have been done on the current macrocyclic series. Simulations were primarily explored by Le Roux et al.,^109^ with five times 100 ns cMD on seven macrocycles. However, its likely hindered by macrocycle-specific barriers of the conformational changes in limited cMD trajectories summarized in the Introduction. Resulted intramolecular hydrogen bond patterns from the incomplete sampling assumed by the authors^109^ were still distant from the PAMPA indications. A follow-up work by Seep et al.^64^ combined short simulations from diverse starting points generated by different software to have a glimpse at the entire conformational space of selected macrocycles in the series, which start to show interesting conformational aspects like the similarity between distributions in water and in DMSO. In our current study, with the help of our previously established protocol^110^ based on aMD,^82^ one single trajectory unifies energy surfaces separated by large energy barriers and only accessible with separated cMD calculations from different starting points previously. Smooth and frequent transitions among individual energy minima within a single trajectory allow direct comparison of free energy levels within the entire conformational space. This results in converged trajectories from different starting points and limits imbalanced sampling. With more converged trajectories independent of starting structures for the whole series, we not only confirm tendencies from previous studies with more clear evidence but also enabled more stable statistical output, which reflects the experimental PAMPA variations with hydrogen bond donor inhibitions.

Here, we test reproducibility of density distribution and transferability of our previously published workflow^110^ with the entire set of 47 systematically modified macrocycles from Le Roux et al.^109^ (Figure 1). In addition to side chain modifications present in our previous study,^110^ this data set also includes challenging multiple backbone variations. Trajectories in water, DMSO, and chloroform together with mechanistic intramolecular hydrogen bond analyses allow the investigation of the solvent effects. We also study the cause and solution for rare convergence issues encountered in various solvents. Finally, the effect of linker length variations and intramolecular hydrogen bond donors are studied in detail, as a proof of concept for a mechanistic view that can be extracted from structural dynamic studies of the macrocyclic series.

### Sampling with aMD Converges and is Independent of the Starting Conformation

We first tested the convergence within single trajectories with 2D RMSD plots (Figure 2A). The excessively sampled conformational switches as well as the stabilized frequency of such events within the trajectories demonstrate homogeneity of conformation distributions sampled at various time intervals. The convergence is further supported by intramolecular contacts and PCAs from split trajectories. Robustness of the workflow with different starting conformations is further demonstrated with the PCA of trajectories starting from different starting structures originating in dissimilar parts of the configurational space (Figure 3). The most challenging macrocycle in our data set for partial charge assignment is macrocycle 35. Even in this case, we show that by averaging partial charges from several ETKDG conformations,^58^ our aMD protocol provides a reproducible free energy surface from different starting conformations and initial velocities, that is, the sampling also converges among trajectories with different starting points.

### Effect of Polar Solvents Mainly Comes from the Dielectric Constant Rather than the Hydrogen Bond Properties

The similarity of the conformational space covered in water and in DMSO (Figure 3) suggests that the effects of polar solvents mainly come from their dielectric constant rather than their abilities to form hydrogen bonds with the macrocycles. Both solvents share a high dielectric constant but differ drastically in their ability to act as hydrogen bond donors. Dampening of the electrostatic interactions and weakened long-range IMHBs seem to be the major driving forces toward less restrained hydrogen bond patterns leading to the “open” conformation (Figure 4B).

Our observation of a similar conformational distributions for the same protonation state in different polar solvents is in line with independent NMR analyses.^139,140^ However, there might be finer differences between the solvents hidden in statistical noise introduced by enhance sampling. Also, more detailed differences might not be easily accessible with large scale statistical analyses and may only be captured by careful inspections of an individual system. The major differences in conformational distributions of peptidic macrocycles seem to come from dielectric constants, although individual solvent properties like hydrogen bond donor/acceptor abundancy may introduce subtle changes.^140^

Surprisingly, there are only marginal differences in the free energy surfaces of macrocycles with different protonation states in water. Although, there are population shifts among or even within major clusters, the conformational space coverage and location of major clusters overlap before and after protonation. The protonated secondary amine group bordering the linker does not seem to favor more interactions with water over IMHBs than with its neutral analogue. The similarity of the conformational spaces sampled among trajectories in different polar solvents and protonation states could already be seen from shorter simulations of selected macrocycles of the series by Seep et al.^64^ Here, with even more thorough enhanced sampling, rather than partial overlaps observed in the previous study, we see complete overlap of the conformational spaces in polar solvents across the protonation states.

### IMHBs Dominate the PCA Changes

Intramolecular hydrogen bond patterns provide a more mechanistic view of conformational distributions (Figure 4). The patterns are rather similar for all simulations in polar solvents, dominated by easily reachable short-range IMHBs, while trajectories in apolar CHCl_3_ are substantially different. DMSO only marginally increases long-range intramolecular hydrogen bond formations compared to water, leaving the conformational landscapes almost identical. The dampening of electrostatic interactions in polar solvents, which massively diminishes long-range intramolecular hydrogen bond formation seems to be the major determinant of the intramolecular hydrogen bond patterns and the subsequent conformational distributions. Meanwhile, additional charge on the hydrogen bond donor D4 in the protonated state is slightly more attractive than its neutral form and increases related intramolecular hydrogen bond formation. This effect is relatively more visible for more rigid molecules with shorter linkers, where the structural rearrangements for intramolecular hydrogen bond formation is more difficult. These mild shifts in intramolecular hydrogen bond patterns result in slight changes in the density distribution of the free energy surfaces. Consequently, these density changes caused by the protonation fade with longer linkers. With both the free energy landscape and intramolecular hydrogen bond formation patterns in polar solvents resembling each other, our results suggest similar conformational distributions in DMSO and thus measurement of the conformational space in DMSO as a substitute for water.

On the other hand, the systems in chloroform are drastically different. Just as the conformational spaces in CHCl_3_ differ from their polar solvent analogues (Figure 3), IMHBs adapt to other preferences in the apolar solvent (Figure 4). Due to the lower dielectric constant in chloroform, the effects of electrostatic interactions get less dampened, that is, amplified compared to the polar solvents. In consequence, long-range IMHBs are significantly promoted in chloroform compared to the polar solvents, opening up more possibilities for intramolecular hydrogen bond formations. Different long-range IMHBs are favored according to geometrical preferences of the macrocycles. Thus, CHCl_3_ results in distinctive intramolecular hydrogen bond patterns and more individual conformational spaces/density distributions, rather than a simple and systematical boosting of the set of IMHBs observed for polar solvents.

### Match between aMD and the Experimental Data

Intramolecular hydrogen bond donor contribution from aMD matches experimentally measured PAMPA trends (Figure 5). Intramolecular hydrogen bond changes in the polar and apolar phases were linked to chameleonicity and cell permeability. Formation of an intramolecular hydrogen bond in apolar phases, especially those in addition to the polar phase, promotes membrane crossing.^37,141,142^ In the macrocyclic series investigated, experimental PAMPA results unambiguously identify the unshielded presence of D4 as the major enhancer of cell permeability, as its inhibitions by additional side chains cause the most significant decreases in cell permeability, that is, increased PAMPA–log *P*_e_ values. On the other hand, we observe that in aMD of the stem structure, D4 contributes most to the intramolecular hydrogen bond in the apolar phase and even more importantly to the difference between polar and apolar phases (Supporting Information, Figure 2). So, our aMD analysis of intramolecular hydrogen bond D4 is in agreement with the PAMPA results.

Very surprisingly, the NMR data infer this central role to D1 and show no intramolecular hydrogen bond activity of D4 (Supporting Information, Figure 3). This is especially puzzling as D4 is quite exposed as it is not in a peptidic bond. So, the NMR results would suggest that alkylation of D1 should have the most negative effect on cell permeability. However, the PAMPA results (macrocycle 28) show that methylation of D1 leads to the most positive effect on cell permeability in the series, which is in line with the significant number of unfavorable intramolecular hydrogen bonds to D1 in aqueous phase in our aMD results.

In summary, the PAMPA results and the NMR results disagree. Our results are in line with the PAMPA results and suggest revisiting the NMR results to investigate why there is no intramolecular hydrogen bond contribution found to D4.

### Sampling Issues with Particular Structural Features

Although the conformational distribution in CHCl_3_ is more informative, it is also more challenging to sample. Lower electrostatic dampening enhances the dense and stable intramolecular hydrogen bond formations, which slow the sampling. This challenge can already be noticed with the 2D RMSD reflecting rigidified behaviors (Figure 2B) and high energy barriers among separated clusters on the free energy surface (Figure 3). These issues are especially pronounced for a subset of the series investigated. We found for molecules with heavy N-alkylation and stereo inversions that hydrogen bond donors/acceptors hinder the intramolecular hydrogen bond exchanges; that is, they slow down the conformational changes and consequently also the convergence of the sampling. Macrocycle 35 is the best example of such a hindered system (Figure 5). It has a combination of N-methylation and stereo inversion. Thus, conformations with IMHBs usually encounter spatial challenges with the bulky groups clashing into each other and the intramolecular hydrogen bond partners found on different sides of the ring prolonging sampling time needed for IMHBs to exchange. It is the only macrocycle in the series that did not pass the reproducibility test with a second initial structure for our initial protocol with partial charges calculated from one single initial structure.

### Macrocycle 35 Convergence Issue (Figure 6) and The Average Charge Approach (Figure 7)

A high free energy conformation can result in unreasonable charges hindering conformational changes and challenging sampling convergence, especially in CHCl_3_. Such a high free energy conformation captured by the randomly generated initial structure 1 for macrocycle 35 seems to have resulted in convergence failure in CHCl_3_ (Figure 6). Sampling with partial charges derived from a more reasonable conformation (initial structure 2), even converges when starting from initial structure 1. In contrast, sampling of more electrostatic dampening aqueous systems converges with charges derived from the two initial structures. Hence, effects of partial charge fluctuations are more emphasized in the apolar solvent with lower dielectric constant.^143^ As a consequence, inaccuracies in partial charges resulting from a very unlikely conformation where the polar NH group in Phe approaches Cα impede the convergence for macrocycle 35. We show that for our model systems this issue can be solved by averaging partial charges from up to 10 conformations. In this way, the inaccuracy in single high free energy conformations get neutralized with corrections from most other reasonable structures and fluctuations cancel out with each other. The average partial charges stabilize at a physically relevant level which allow even macrocycle 35 in CHCl_3_ to converge (Figure 7, Supporting Information, Figures 8 and 9). With average charge, all free energy surfaces from macrocycle 35 converge in multiple tests with different initial structures.

### Linker Effects

Following our workflow, we find that the linker mainly controls the flexibility of the investigated macrocycles in water and polar solvent (Figure 8). PCA plots for aqueous systems are in line with the intramolecular hydrogen bond patterns for a systematic increase of flexibility as the linker grows. The low free energy area of the conformational space radiates to surrounding areas consistently as the linker length increases. However, the shape of the overall space sampled or transitions within the space are scarcely changed along the series. Accordingly, the intramolecular hydrogen bond patterns show a systematic increase in the amount of the same set of short-range IMHBs throughout the series. This reflects the lack of conformational preferences in polar solvents throughout the series, longer linkers merely seem to render the molecule more flexible and increase the chance of reaching nearby conformations.

On the other hand, linker length changes both the flexibility and the geometry of the macrocycles in the apolar solvent. Although the free energy surfaces also extend with the linker length in chloroform, distinctive conformational space preferences and high free energy barriers separating the conformational islands indicate the formation of unique structural clusters along the series. Indeed, the contact maps show individual intramolecular hydrogen bond patterns. Meanwhile, all CHCl_3_ patterns favor more long-range IMHBs than their aqueous analogues, in line with the hiding of polar surfaces in the “closed” conformations of the chameleonic behavior. However, shifts in the preference of the long-range IMHBs throughout the series reflect the individual changes in geometry of the macrocycles for each linker length. This implies that different from very similar conformational distribution in polar solvents, in apolar environments macrocycles adapt more distinctive and constrained conformations. In conclusion, this suggests that the conformational distribution in the chloroform might be the major difference for the membrane crossing properties.

## Conclusions

We find that our previously described aMD protocol is efficient and can be extended to a large set of macrocycles. With reasonable partial charges, it converges for each macrocycle in a set of 47 systematically modified macrocycles with highly diverse modifications in polar and apolar solvents without prior structural knowledge. Convergence of our approach is validated by comparing free energy surfaces obtained using different starting structures for all macrocycles.

We observe distinct structural constraints in polar and apolar solvents, conformational changes of macrocycles in polar solvents are more systematic, while those in chloroform are less predictable. Conformational ensembles sampled in water and DMSO are surprisingly similar. For example, for the linker series, the cluster simply extends without shifting among clusters. It indicates that effects of the polar solvents mainly originated from their high dielectric constants and dampening of the electrostatic interactions. Although subtle differences may still be revealed by careful individual inspection in further studies, especially for much larger molecules with more contacts to solvents. Additionally, we also observe similar conformational spaces and limited population shifts among/within major clusters for positively charged and neutral species in water, which can be explained by the mild influence of the protonation of the ring amine on intramolecular hydrogen bond formation in a polar environment. Overall, systems in polar solvents are dominated by short-range intramolecular hydrogen bonds among the neighboring residues. Although flexible molecules have more chance to form those intramolecular hydrogen bonds, their conformational spaces only extend systematically as the linker grows, with their conformational preferences staying similar. However, the systems in chloroform are more variable. For example, at each linker length, the conformational distribution adapts to individual preferences. As can be seen with intramolecular hydrogen bond patterns, the low dielectric constant of chloroform lowers the electrostatic dampening, which promotes more stable and energetic long-range intramolecular hydrogen bonds. These long-range intramolecular hydrogen bonds are strongly dependent on the geometry of the macrocycle and change unexpectedly from one pattern to another as the linker grows. Because the chloroform conformations are much more dissimilar than those in polar phases, they likely make the major differences for membrane crossing.

Although the chloroform conformations are more informative for membrane-permeable molecules with chameleonic behaviors, they are hard to sample. As the dielectric constant drops, energy barriers to alter intramolecular hydrogen bond formation become more significant, slowing down the conformational changes. Meanwhile, as the electrostatic interactions get less dampened, the effect of the partial charges becomes more important. We show that an individual random conformation in a high free energy state can hinder the sampling in the apolar phase and damage the convergence. However, this can be fixed by averaging up to 10 conformations to dilute the inaccuracy introduced by rare conformations.

To sum up, we evaluate the aMD protocol for macrocycle conformational sampling and explore its application in understanding the effects of structural modifications on cell membrane permeability. Samplings in various solvents and protonation states highlight the role of conformational distribution in apolar solvent for membrane crossing. However, the convergence in apolar solvent is more problematic, and we recommend the averaged partial charges to reduce inaccuracy in this low dielectric constant environment with less electrostatic dampening.

## Summary

We evaluate and extend a previously established macrocycle conformational sampling workflow to a large systematically modified peptidic macrocyclic series from Le Roux et al.^109^ The systematic structural changes enable a thorough comparison throughout the entire series, while the large variety of modifications covered intrinsically test the validity of a sampling protocol. We apply accelerated MD to address hindrance in macrocyclic samplings, such as the exchanges among dense hydrogen bonds and cis–trans conversion of the peptidic bonds. We validate the convergence of samplings from the workflow and investigate them mechanistically in the presence of IMHB patterns. We find that effects of polar solvents are dominated by their dielectric constants and the conformations in apolar environments are more variable along the series, thus crucial for comparisons among macrocycles. We reaffirm that using averaged partial charges arising from several conformations ensures sampling efficiency in the apolar phase, where the macrocycle conformation sampling is decelerated by less dampened electrostatics. Thus, our study provides suggestions to address macrocycle samplings and adds a mechanistic view of conformational distributions in polar and apolar environments.

## Data Availability

The Cartesian coordinates and the partial charges of the starting structures as well as cluster representatives discussed in this paper are available in the Supporting Information as a ZIP file. The initial structures are generated by RDKit, the RDKit, and ETKDG v3 module can be purchased at https://www.rdkit.org/and https://www.rdkit.org/docs/source/rdkit.Chem.rdDistGeom.html. The protonation was done with MOE, MOE can be purchased at https://www.chemcomp.com/Products.htm. Gaussian 09 was used for the partial charge calculation calculations. Gaussian software can be purchased from https://gaussian.com/. The systems were prepared using AMBER20 software. The AMBER package can be purchased at https://ambermd.org/index.php. The systems were analyzed with AmberTools 19, AmberTools can be purchased at https://ambermd.org/AmberTools.php. PyMOL v2.3.0 were used for visualization and analysis of the system. PyMOL can be downloaded from https://pymol.org/.

## Supporting Information Available

Explanation of short-range IMHB, contact plots of IMHB derived from NMR and aMD, distribution of ETKDG structures used for charge generation, *B*-factors of atoms involved in the PCA in dependence of linker length, time dependence of sampling in PCA space, PCA plots resulting from trajectory splitting for macrocycle 35, time dependence of omega angles (PDF)
Starting structure for macrocycle 35, protonated structures for all possible macrocycles, and PDB files of cluster representatives for the two clusters from various macrocycle 1 systems in Figure 3 (ZIP)
