# Supplementary material for: Addressing Challenges of Macrocyclic Conformational Sampling in Polar and Apolar Solvents: Lessons for Chameleonicity
Source: J Chem Inf Model. 2023 Nov 9;63(22):7107–23. doi: 10.1021/acs.jcim.3c01123 (PMC10685455; doi:10.1021/acs.jcim.3c01123)
Supplement: Supplementary file 1 — ci3c01123_si_001.pdf [file ci3c01123_si_001.pdf]

Supporting Information

Addressing challenges of macrocyclic conformational sampling in polar and apolar solvents – lessons for chameleonicity

Xuechen Tang[†], Janik Kokot[†], Franz Waibl[†,‡], Monica L. Fernández-Quintero[†], Anna S. Kamenik[‡], Klaus R. Liedl*[,†]

† Department of General, Inorganic and Theoretical Chemistry, University of Innsbruck, A-6020 Innsbruck, Austria

‡ Department of Chemistry and Applied Biosciences, ETH Zürich, 8093 Zürich, Switzerland

Email: Klaus.Liedl@uibk.ac.at

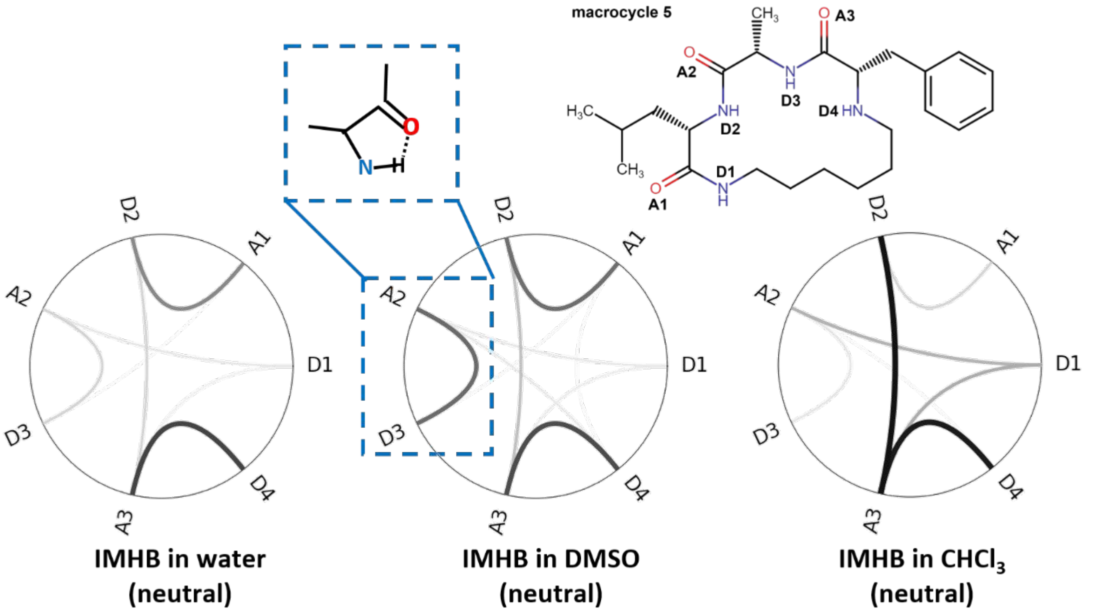

SI.Figure 1. Intramolecular hydrogen bond (IMHB) contribution of individual hydrogen bond donors of the reference scaffold (macrocycle 5) in polar and apolar solvents.

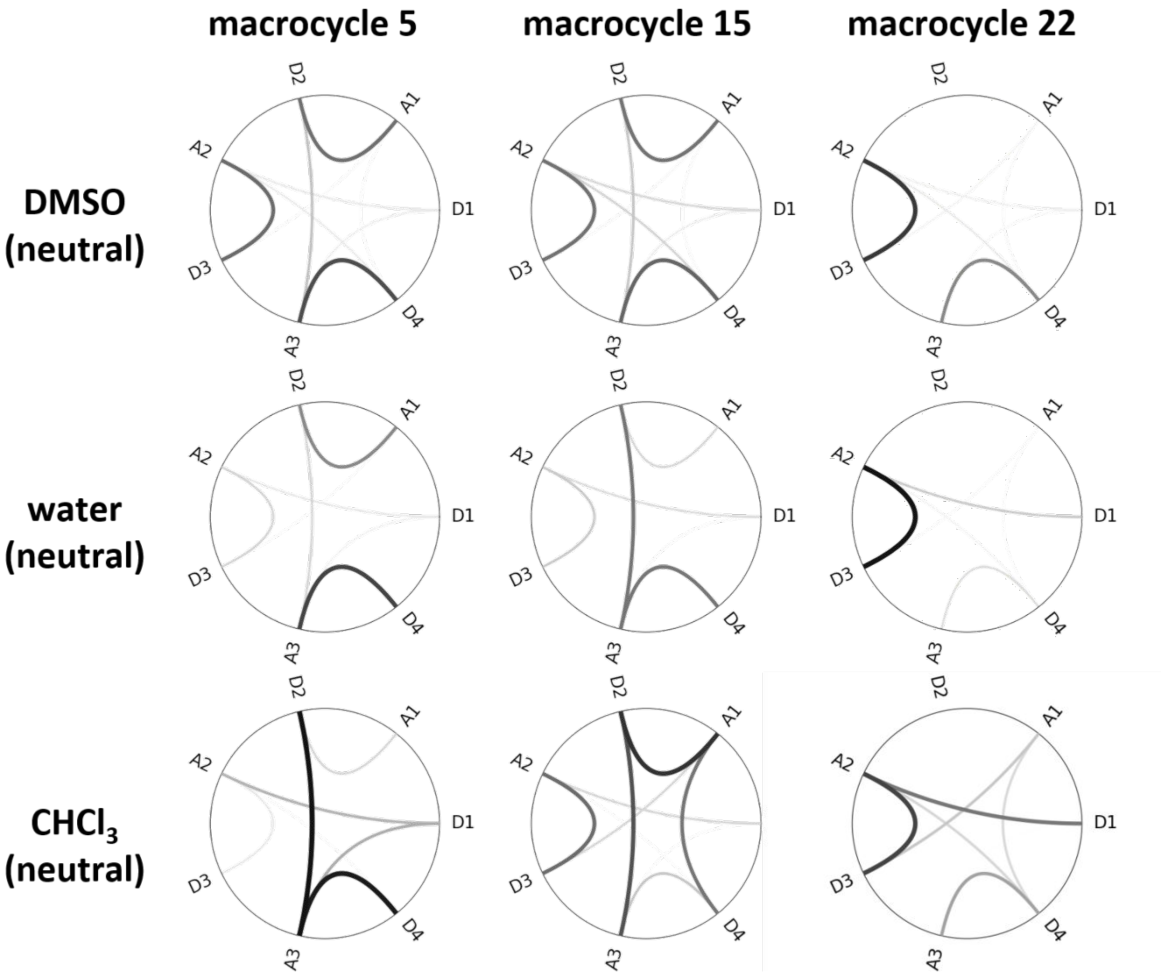

SI.Figure 2. Sampled IMHB patterns for systems originally measured by NMR in the experimental report[1].

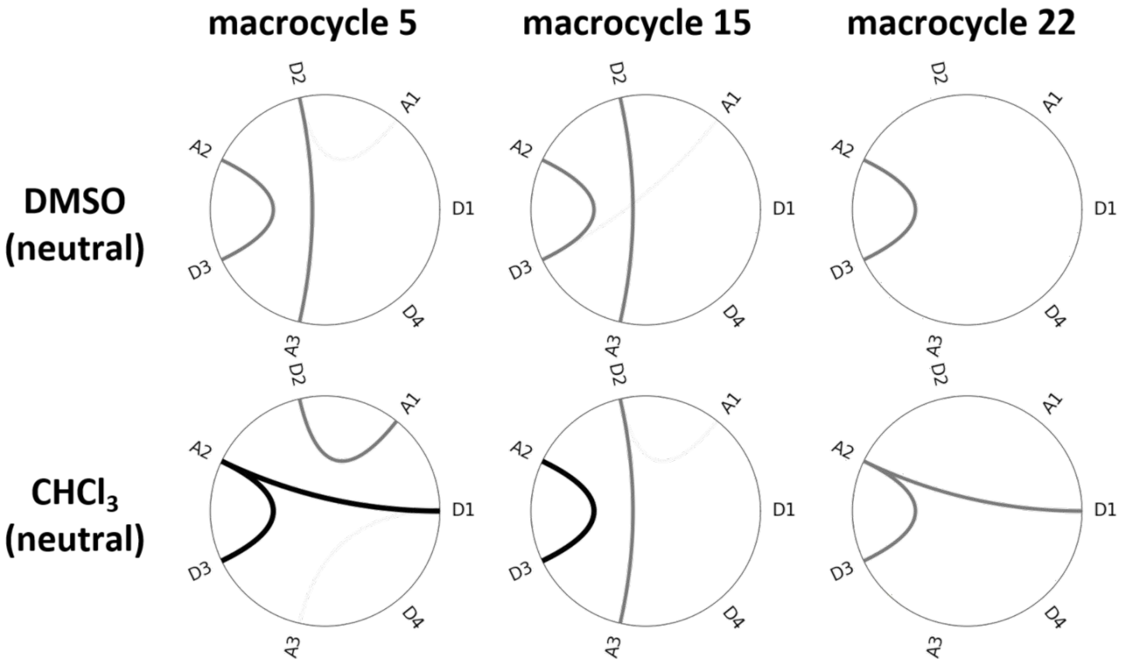

SI.Figure 3. Contact maps deduced by NMR from le Roux et al.[1] *Surprisingly, they do not show the prominent hydrogen bond donor D4 bordering the linker, which is not constrained by peptidic bond.*

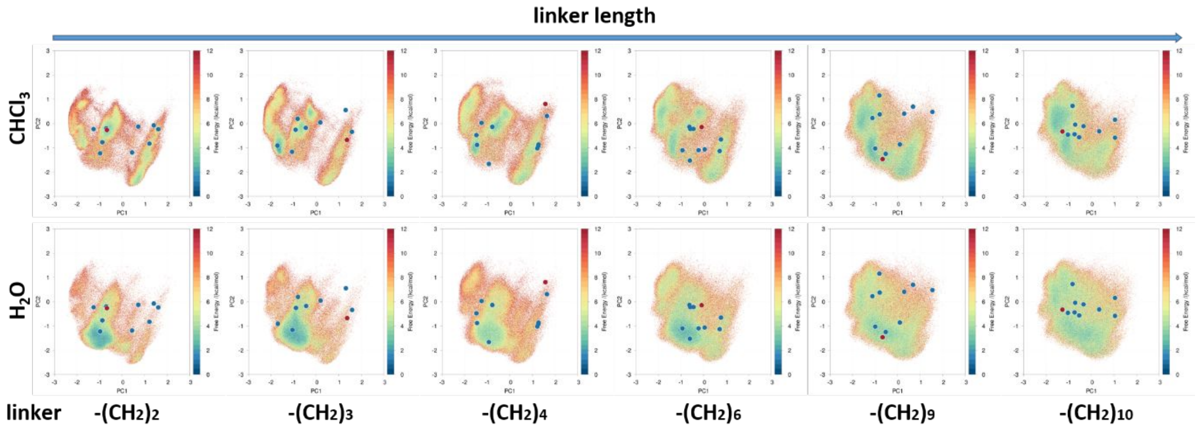

SI. Figure 4 PCAs with ETKDG structures used to generate the average charges for Figure 7. Starting structures for samplings are marked with red dots while those only contribute to partial charge calculations are marked in blue.

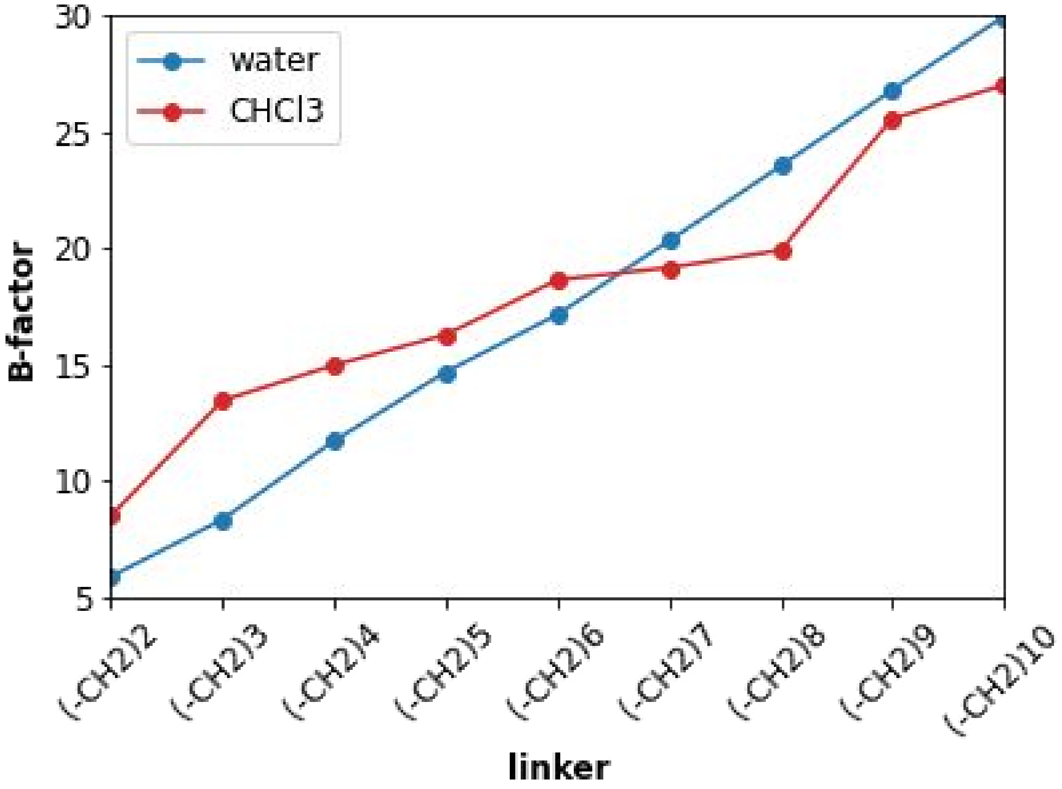

SI.Figure 5 Weight averaged B factors of atoms involved in the PCA for the linker series: (both water and chloroform show upward trend)

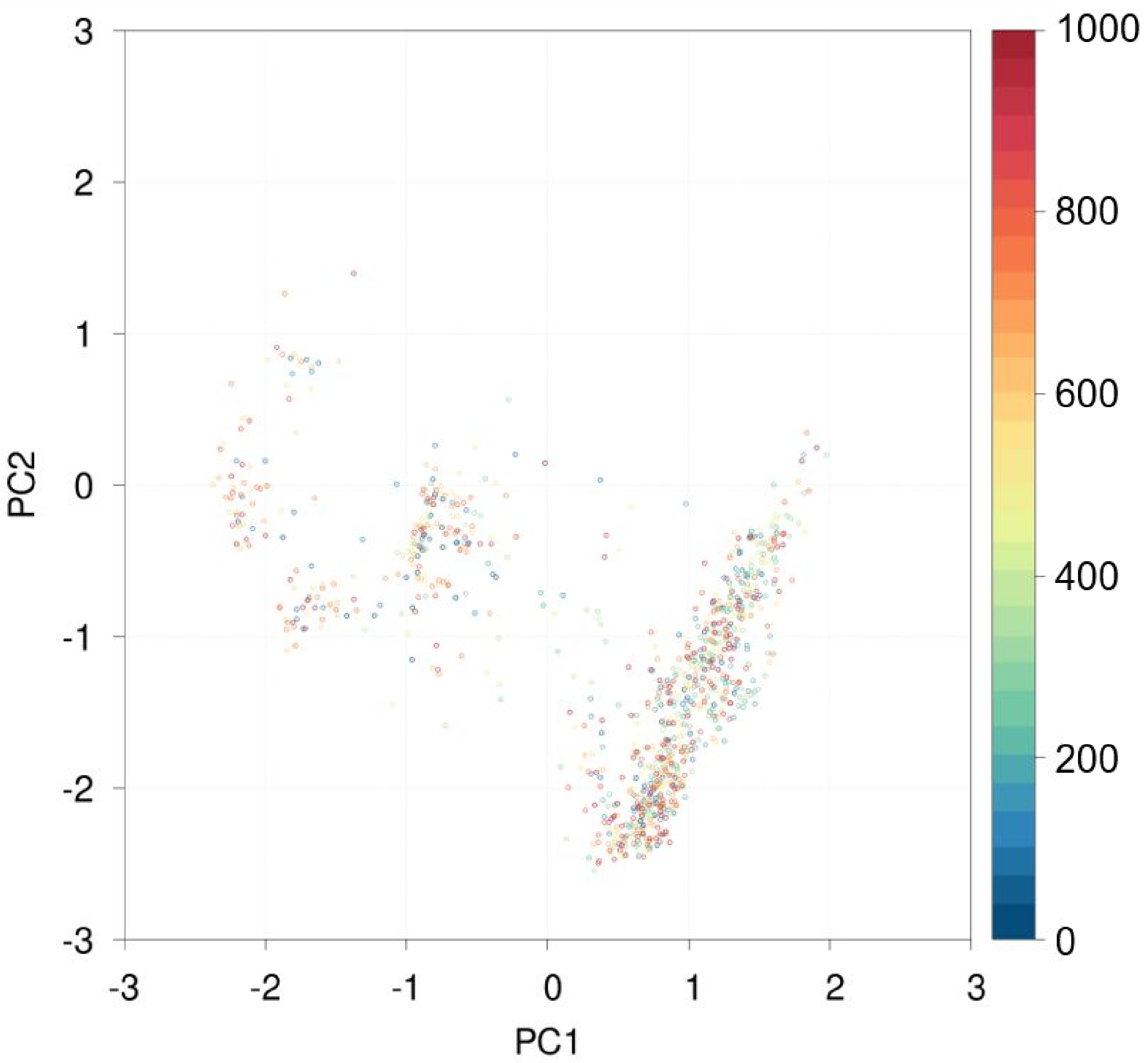

SI.Figure 6 PCA of macrocycle 1 with the shortest linker (-(CH_2)$_2$) in CHCl3, colored by frame number (1000 frames = 1µs aMD, same as 2D RMSD). Projections from different time points are homogeneously distributed in the conformational space sampled by the entire trajectory.

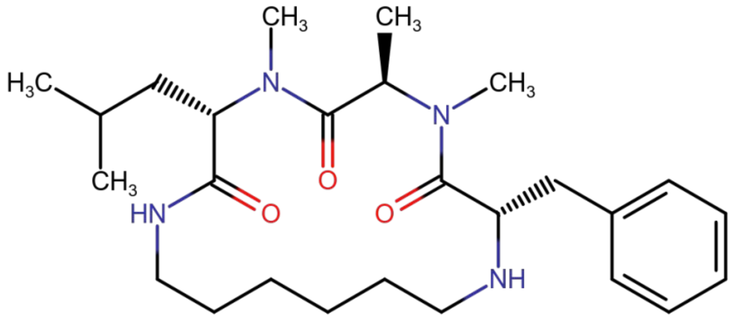

SI.Figure 7 2D representation of the macrocycle 35.

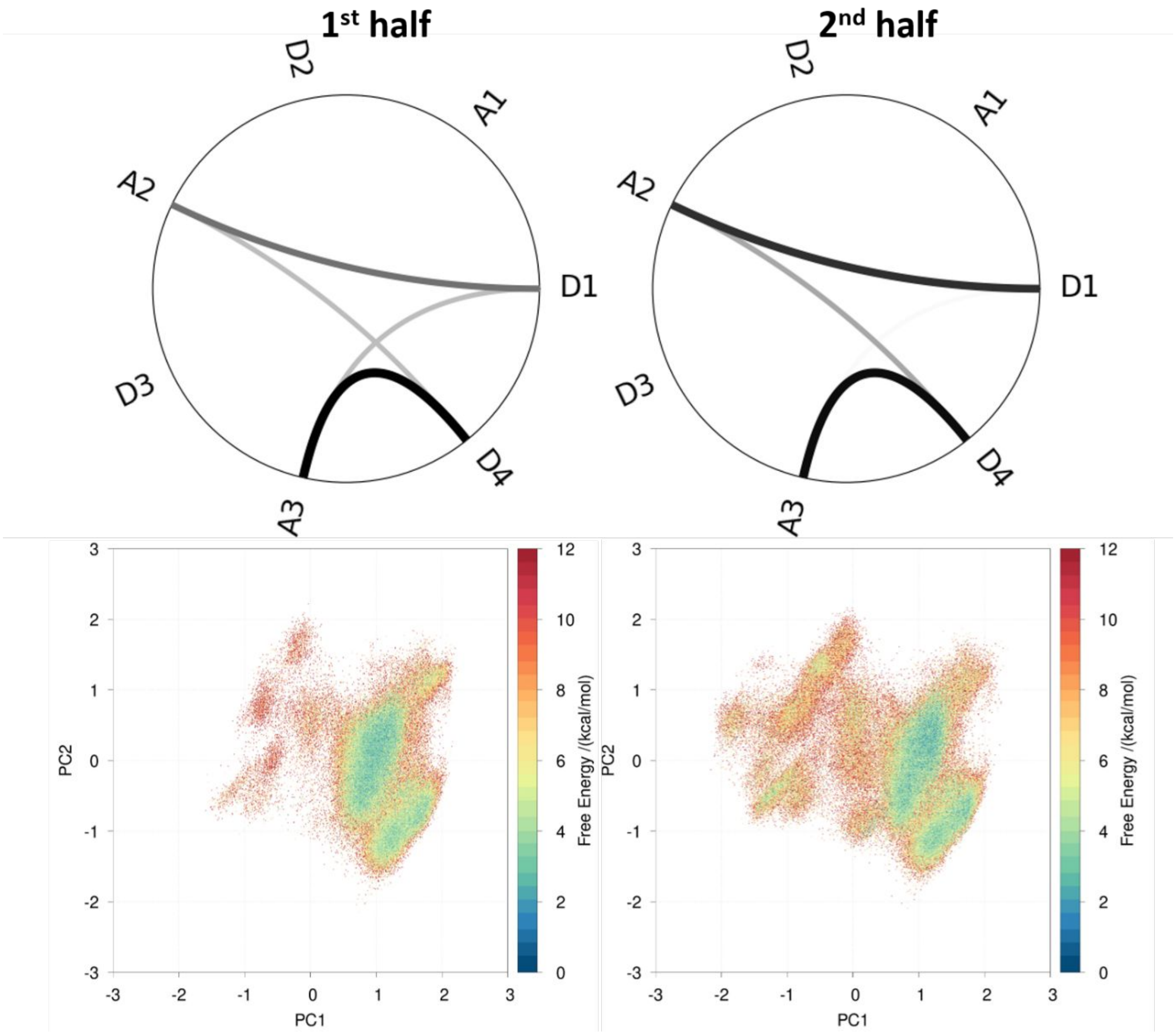

SI.Figure 8 Macrocycle 35 with average charges in chloroform shows good convergence. Complementing convergence among trajectories from different starting structures, we also observe coherence between two halves of the split single trajectory. The contact maps agree on major contacts with limited fluctuation of frequency. Similarly, PCAs of the splits agree on major clusters with fluctuation on sampling frequency of high free energy clusters.

Omega of macrocycle 1 RESP model:

Leu-linker

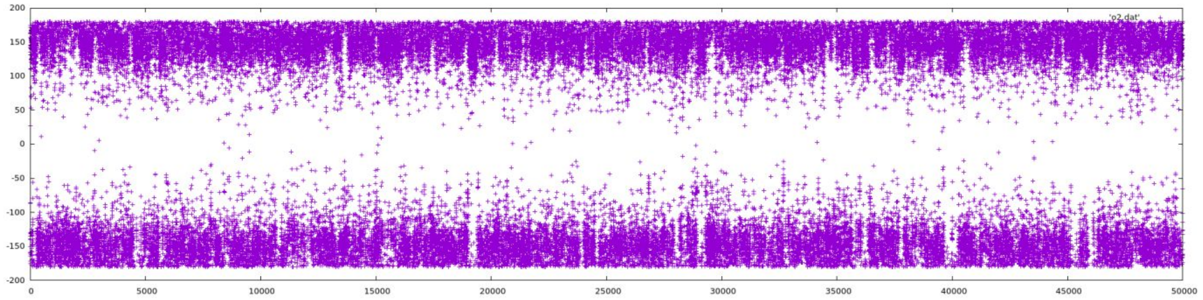

Leu-Ala

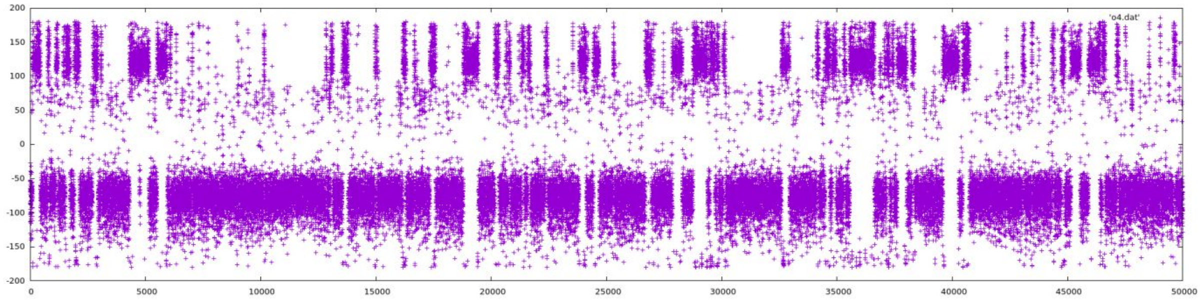

Ala-Phe

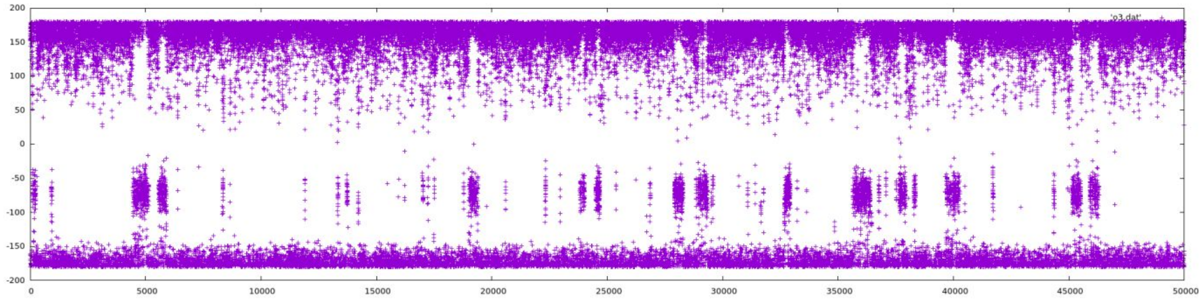

Omega of macrocycle 35 RESP model:

Leu-linker

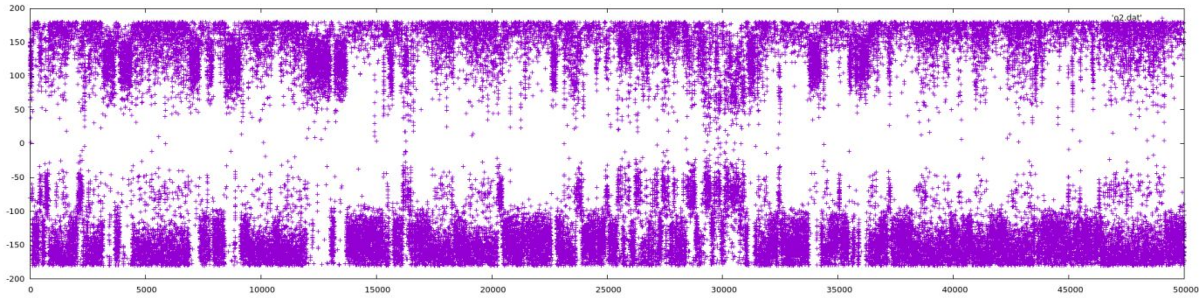

Leu-Ala

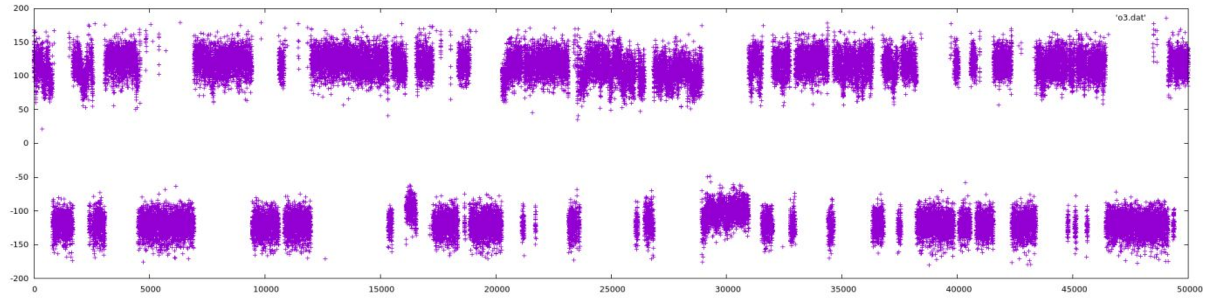

Ala-Phe

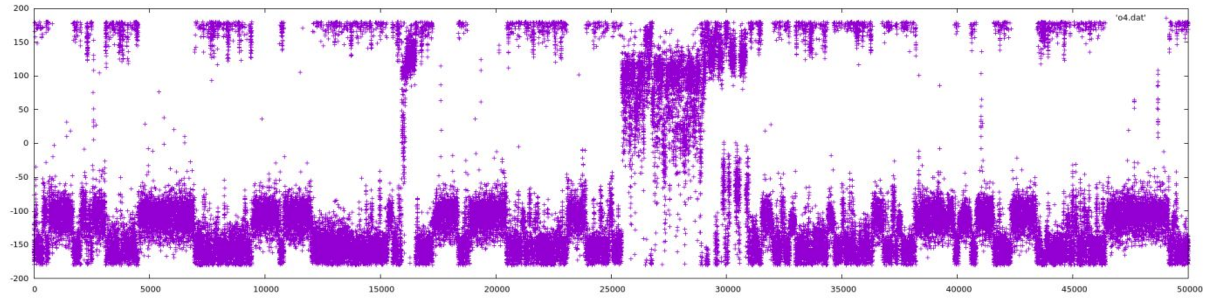

Omega of macrocycle 35 AM1-BCC model:

Leu-linker

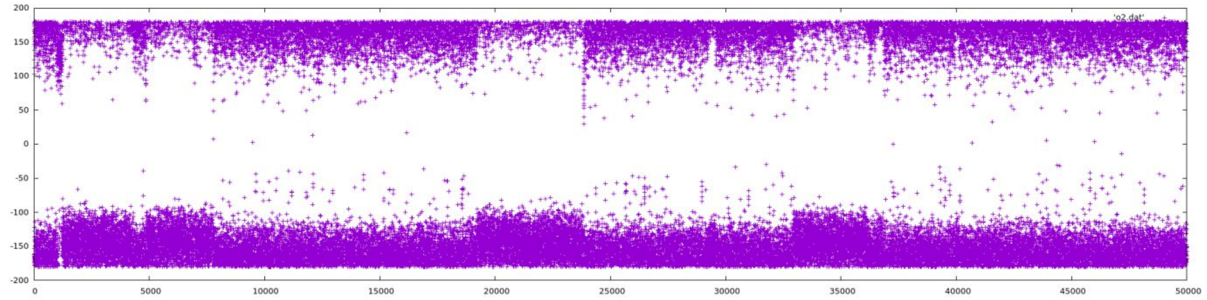

Leu-Ala

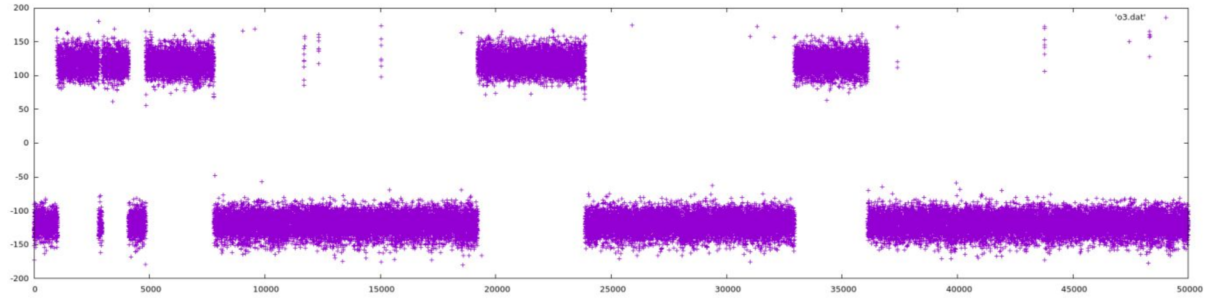

Ala-Phe

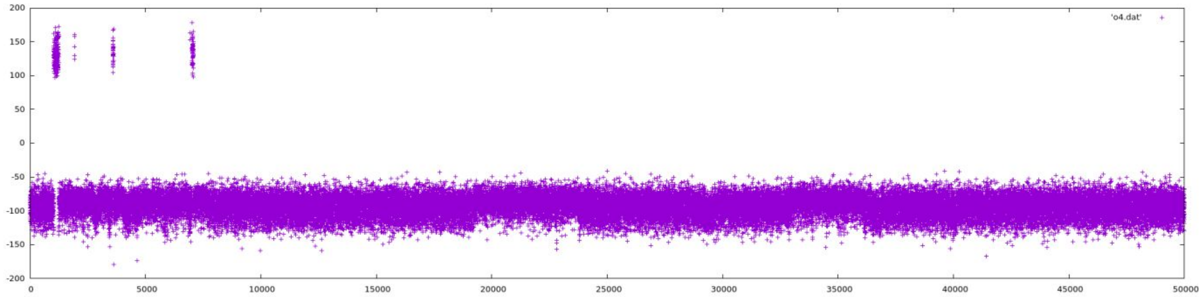

SI.Figure 9 The cis-trans conversions in most challenging systems and with different partial charge models. Macrocycle 1 and 35 in chloroforms (even more in water) generally show frequent transitions with RESP[2] model, but it can be rarer in less variable AM1-BCC[3] models. Note that angles between 0 and 180 degrees are plotted by high energy transition states and will be downweighted during reweighting.

References:

(1) Le Roux, A.; Blaise, É.; Boudreault, P.-L.; Comeau, C.; Doucet, A.; Giarrusso, M.; Collin, M.-P.; Neubauer, T.; Kölling, F.; Göller, A. H.; Seep, L.; Tshitenge, D. T.; Wittwer, M.; Kullmann, M.; Hillisch, A.; Mittendorf, J.; Marsault, E. Structure-Permeability Relationship of Semipeptidic Macrocycles-Understanding and Optimizing Passive Permeability and Efflux Ratio. *J Med Chem* **2020**, *63* (13), 6774–6783. https://doi.org/10.1021/acs.jmedchem.0c00013.

(2) Bayly, C. I.; Cieplak, P.; Cornell, W.; Kollman, P. A. A Well-Behaved Electrostatic Potential Based Method Using Charge Restraints for Deriving Atomic Charges: The RESP Model. *J. Phys. Chem.* **1993**, *97* (40), 10269–10280. https://doi.org/10.1021/j100142a004.

(3) Jakalian, A.; Jack, D. B.; Bayly, C. I. Fast, Efficient Generation of High-Quality Atomic Charges. AM1-BCC Model: II. Parameterization and Validation. *J Comput Chem* **2002**, *23* (16), 1623–1641. https://doi.org/10.1002/jcc.10128.
